# A topography-sensitive 2SFCA approach to measuring accessibility to active living infrastructure and its association with health-enhancing physical activity

**DOI:** 10.3389/fpubh.2026.1923137

**Published:** 2026-09-04

**Authors:** Yesong Shin, Jeongwoo Lee

**Affiliations:** Department of Urban Design and Studies, College of Engineering, Chung-Ang University, Seoul, Republic of Korea

**Keywords:** active living infrastructure (ALI), composite accessibility index, health-enhancing physical activity (HEPA), international physical activity questionnaire (IPAQ), topographic vulnerability, topography-sensitive 2SFCA (T-S2SFCA)

## Abstract

**Introduction:**

Pedestrian accessibility to neighborhood infrastructure is associated with Physical Activity (PA), yet the built environment is often evaluated as separate facility types, while accessibility measures may not jointly capture population demand and topographic walking burden. This study therefore examined PA in relation to a composite Active Living Infrastructure (ALI) measure that integrates five facility types and applies a Topography-Sensitive Two-Step Floating Catchment Area (T-S2SFCA) model, which weighs facility supply against population demand while adjusting walking distance for slope.

**Methods:**

The model was applied to examine the relationship between pedestrian accessibility to neighborhood facilities and multilevel PA behavior among adult residents in Seongnam, South Korea. Five types of neighborhood amenities—retail, community, public green spaces, public transit, and sports facilities—were conceptualized as a single interdependent ALI system supporting daily mobility, leisure, and structured exercise. Using the proposed T-S2SFCA model, spatial accessibility was calculated across 1,580 residential grid cells at a high-resolution 250 m × 250 m scale. The resulting indices were then linked to cross-sectional survey data (*N* = 555) and analyzed using multinomial logistic regression across activity levels derived from the International Physical Activity Questionnaire (IPAQ).

**Results:**

The empirical findings revealed distinct patterns of association between facility-specific accessibility and PA levels. ALI components were associated with PA at different activity levels. Public green spaces and retail facilities were associated with participation in both activity levels. By contrast, sports and community venues were associated exclusively with Health-Enhancing Physical Activity (HEPA). When integrated, the composite ALI accessibility index exhibited robust positive associations with both minimally active and HEPA levels.

**Conclusion:**

These results support the systemic conceptualization of ALI and suggest that a neighborhood’s capacity to accommodate active living is associated with the synergetic composition and functional complementarity of multiple infrastructure elements working together as a whole rather than with the separate aggregation of isolated destinations. These findings suggest the value of a shift in healthy city planning, away from baseline proximity quotas for individual facilities and toward the coordinated development of cohesive ALI networks that support routine PA across complex terrains.

## 1 Introduction

Physical activity (PA) plays a pivotal role in the prevention of chronic diseases and the promotion of health; however, a substantial proportion of adults still fail to meet the recommended levels of PA (1, 2). PA cannot be explained solely by individual-level factors, and it is shaped by the physical conditions of the neighborhood environment in which daily life unfolds (3–5). Therefore, understanding how the neighborhood environment provides opportunities for PA requires attention not only to a single facility, but also to the broader set of resources that support residents’ everyday movement, leisure, and structured exercise.

This study defines Active Living Infrastructure (ALI) as a concept encompassing neighborhood facilities that support residents’ PA, including public transit, public green spaces, and retail, sports, and community facilities. These five facility types were combined because they represent complementary domains of residents’ daily activity environments: retail facilities and public transit support routine mobility, public green spaces support leisure walking and exercise, and sports and community facilities support structured and program-based activities. An environment that supports PA cannot be explained solely by the accessibility of a single facility type; rather, it is necessary to consider multiple dimensions of accessibility that collectively support daily mobility, leisure activities, and structured exercise (6–8). Accessibility to retail facilities has been highlighted as a neighborhood environmental factor that enhances the potential for purposeful daily travel and walking activities (9, 10), and public transit has been reported to increase daily walking volume during the process of walking to and transferring at transit stops (11, 12). Accessibility to public green spaces has been examined as a representative environment supporting PA that provides opportunities for leisure walking and exercise (13–16), and accessibility to sports facilities and community facilities can be associated with participation in structured exercise, program-based activities, and community-based outing opportunities (6, 17–19). Conceptualizing these facilities as a single interdependent infrastructure, rather than a collection of separate destinations, provides a basis for evaluating a neighborhood’s overall environmental capacity to accommodate PA.

Measuring ALI accessibility requires accounting not only for facility supply but also for population demand. Existing methods rely on indicators such as the number or density of facilities within a certain area and the distance to the nearest facility; however, they do not sufficiently reflect the fact that, even for the same facility, the relative opportunity for individual residents to use it decreases as the demand from the population increases (20–24). The Two-Step Floating Catchment area (2SFCA) method addresses this by jointly reflecting facility supply and population demand (25), and subsequent extensions have continued to approximate actual usage behavior more closely (26, 27).

Population-demand adjustment, however, has typically been implemented with planar distance, which confines accessibility to two dimensions and leaves topography outside the measure. Slope and elevation differences reduce walking speed and increase the physical burden of walking (28, 29), yet they are rarely embedded in supply–demand-based accessibility measures. This omission is particularly consequential in Korea, where many cities have developed within basins surrounded by mountainous terrain. In such settings, gently sloped living areas and steep residential areas can fall within a single administrative district, so that residents nominally sharing the same neighborhood face markedly different walking conditions. The study area is one such city, with terrain differentiated along a north–south gradient. The northern old town consists largely of sloped land, its interquartile range spanning 5° to 12°, whereas the southern new town is relatively flat, with mountainous terrain confined to its margins. Commercial districts in both areas are generally situated on level ground, but residential areas differ markedly: those in the northern old town are dispersed across terrain ranging from gentle to steep slopes, whereas most residential areas in the southern new town are located on land with slopes of less than 2°.

The relationship between ALI accessibility and PA may differ across individual PA levels. Previous studies have reported mixed findings on the relationships between walking environments, facility accessibility, and PA, which vary with facility quality, user perceptions, and socioeconomic characteristics (30, 31). These mixed results suggest that the relationship between accessibility and PA may differ depending on the level of activity, which a dichotomous approach distinguishing only the presence or absence of activity cannot be captured. The International Physical Activity Questionnaire (IPAQ) addresses this by converting responses into MET minutes per week to classify PA into inactive, minimally active, and Health-Enhancing Physical Activity (HEPA) and has been widely used as a standardized tool (32, 33).

Taken together, these issues point to a need for an analytical framework that integrates composite ALI accessibility, population demand, and topography-sensitive walking costs when examining individual PA levels. Existing studies have typically assessed facility types separately, while accessibility measures have not jointly incorporated population demand and topographic walking burden. To address this gap, this study constructed a composite ALI accessibility measure across five facility types using T-S2SFCA. The model accounts for facility supply relative to population demand while incorporating slope-adjusted walking costs. First, the study examined the association between ALI accessibility and the likelihood of being minimally active or achieving HEPA relative to being inactive. Second, it compared facility-type and composite ALI accessibility in their associations with PA levels and identified which ALI components were most strongly associated with each activity level.

## 2 Methods

### 2.1 Study area

**Figure 1:**
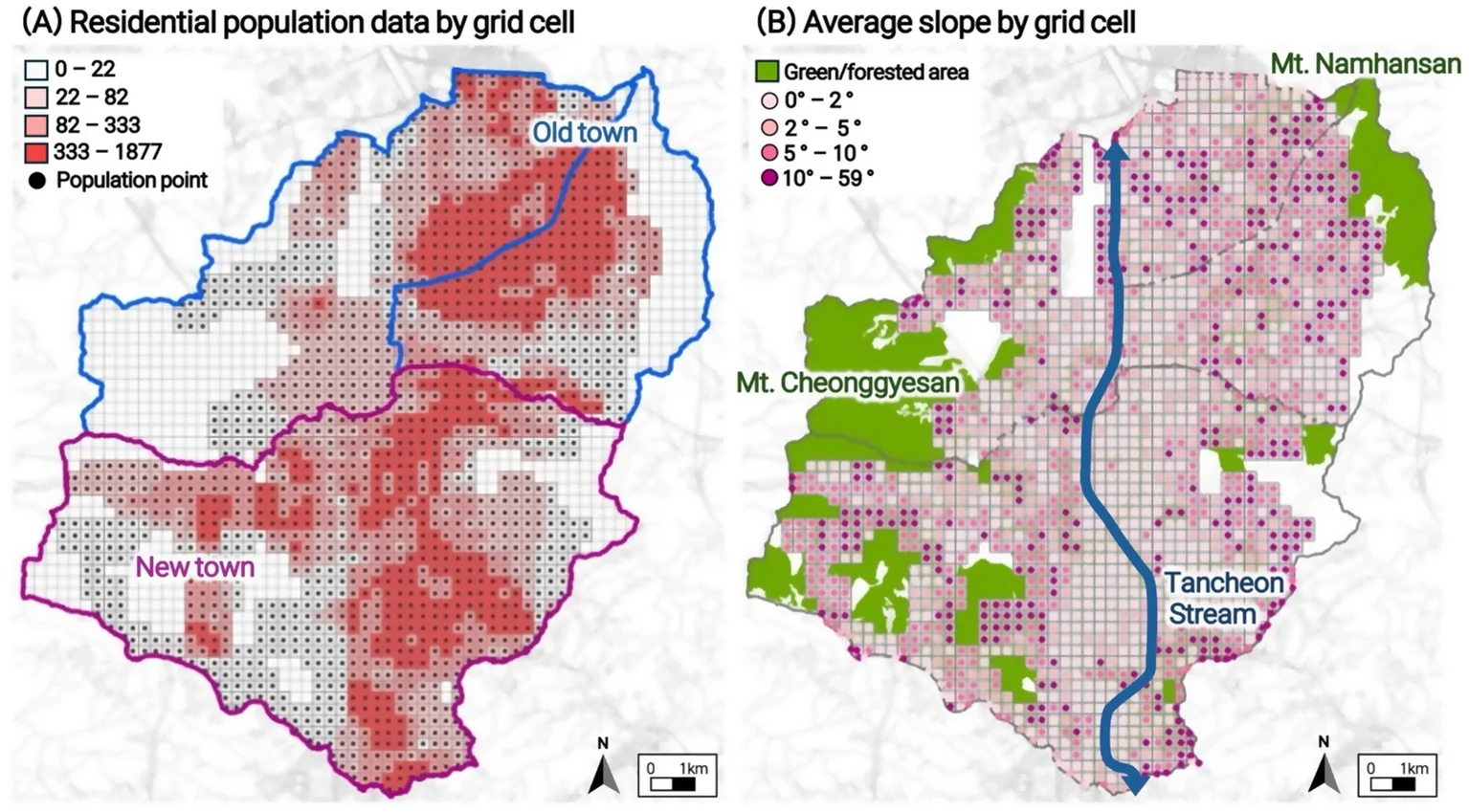
Study area: **(A)** Residential population data by grid cell and **(B)** Average slope by grid cell in Seongnam.

This study was conducted in Seongnam, a city with approximately 900,000 residents, located southeast of Seoul, Republic of Korea. Seongnam was considered an ideal setting for examining topographic vulnerability: its northern old town consists predominantly of sloped residential areas, whereas its southern new town places residential areas on relatively flat land. The two urban districts each have their own central commercial core and road patterns adapted to their respective terrain, yet they are bound together within a single administrative jurisdiction and share the same natural environment—including the Tancheon stream and surrounding mountains. This configuration makes it possible to isolate how differences in topography and urban-planning context, rather than disparate natural settings, shape accessibility within a shared urban context. The median slope of grid cells was 2° in the southern new town and 10° in the northern old town (Figure 1).

### 2.2 Research design

This study used a cross-sectional research design to examine the relationship between ALI accessibility and PA levels among adult Seongnam residents. This design links neighborhood-level walking accessibility, measured while reflecting topographic conditions, with individual PA levels, thereby allowing the examination of how accessibility is associated with different PA levels.

To evaluate this relationship, individual-level data were collected through an online survey. The final analytical sample consisted of 555 adult Seongnam residents. PA was measured using the IPAQ and classified into three levels: inactive, minimally active, and HEPA. Each respondent’s residential address was geocoded and linked to a 250 m residential grid cell containing their residence. Grid-level accessibility was calculated as facility-type accessibility and composite ALI accessibility using the T-S2SFCA model. Each respondent was assigned accessibility values for their residential grid cell.

The analysis was conducted in two stages. First, the accessibility estimates derived from the 2SFCA and T-S2SFCA models were compared across slope intervals to examine how topographic correction altered the spatial distribution of facility-type and composite ALI accessibility. For comparison, the conventional 2SFCA model was estimated using the same supply and demand data, 400 m catchment radius, and distance-decay function, with the only difference being the use of unadjusted network distance instead of slope-adjusted travel distance. Second, multinomial logistic regression models were used to analyze the association between ALI accessibility and PA levels. These models were employed to estimate associations with each activity level separately, rather than assuming that minimally active and HEPA are related to ALI accessibility in the same manner. Inactive status was set as the reference category, and the odds of being minimally active or achieving HEPA were estimated. Model 1 included facility-type accessibility and Model 2 included composite ALI accessibility. All models controlled for sex, age, monthly income, duration of residence, Patient Health Questionnaire-9 (PHQ-9) scores, and life satisfaction. These covariates were included to account for individual sociodemographic characteristics, residential tenure, and psychological well-being that may influence PA independently of neighborhood accessibility.

### 2.3 Data and measures

The study area was divided into 250 m × 250 m grid cells, which were used as spatial units for constructing the facility supply and population demand data. Population demand data were reconstructed into grid cells by applying areal-weight interpolation to the 2024 census output area level population data provided by the Statistical Geographic Information Service of Statistics Korea. This grid resolution maintains spatial detail without oversimplifying the range of daily movements (34–36). Grid cells with no resident population and those located within large green areas were excluded from the analysis to reflect the distribution of mountains and green spaces along the urban peripheries. As a result, 788 of the 2,368 grid cells were excluded, leaving 1,580 residential grid cells for the final analysis, and the centroid of each grid cell was defined as the representative demand point.

Supply data were constructed using POI data representing ALI. Based on previous studies, ALI was classified into five facility types: retail, community, public green spaces, public transit, and sports facilities. Table 1 lists the definitions, data sources, and number of facilities for each type. Facilities located within the administrative boundaries of Seongnam were extracted, and duplicate records were removed prior to analysis.

**Table 1:** Definitions, counts, and data sources of Active Living Infrastructure (ALI) facility types.

Facilities	Description	Number	Source
Retail facility	Facilities that provide core daily convenience goods and services for urban living, including banks, shops, post offices, large retailers, traditional markets, restaurants, convenience stores, and pharmacies.	9,756	V-world / Kakao Map API / Health Insurance Review and Assessment Service / Small Enterprise Distribution Agency
Sports facility	Facilities that promote PA and help maintain muscle strength, balance, and cardiopulmonary function, including public and private exercise-based venues.	1,796	
Community facility	Facilities that support social health through cultural and leisure activities, including welfare centers, community centers, libraries, museums, and religious facilities.	1,955	
Public green space	Facilities that provide opportunities for PA and contribute to psychological stability and social interaction, including neighborhood parks, small parks, riverside parks, and cultural or sports parks.	729	Environmental Spatial Information Service / National Spatial Data Infrastructure Portal / Open Street Map
Public transit	Facilities that enable short- and medium-distance travel within neighborhoods, improving residents’ mobility and accessibility, including bus stops and subway stations.	1,312	OpenStreetMap

A pedestrian network was constructed using pedestrian network data obtained from OpenStreetMap. Only walkable links were extracted and refined based on tags, such as footways and paths. To reflect topographic constraints on pedestrian movement, slope values were calculated from the 5 m resolution DEM provided by the National Geographic Information Institute and assigned as attributes to each network link. Areas without connected walkable links, such as roadless mountainous areas, were treated as inaccessible in the network analysis.

The survey was conducted online among residents of Seongnam between October and November 2024. Participants were recruited through local, community-based online platforms. The study protocol was approved by the Institutional Review Board of the researcher’s institution (IRB No. 1041078-202110-HR-310-0). In total, 620 individuals responded to the survey. After excluding respondents who exhibited insufficient response consistency and those with severe depressive symptoms (PHQ-9 score ≥ 14), the final analytic sample consisted of 555 participants. Respondents with severe depressive symptoms were excluded because their PA levels were likely to be substantially constrained, independent of neighborhood accessibility. PHQ-9 scores were included as covariates in the final models to control for residual depressive symptoms.

PA levels were measured using the IPAQ Short Form, which assesses the frequency and duration of vigorous-intensity activity, moderate-intensity activity, and walking during the previous 7 days. Responses were converted into MET-minutes/week and classified into three categories following the IPAQ scoring protocol (33): HEPA (vigorous activity ≥3 days/week with ≥1,500 MET-min/week, or activity on 7 days/week with ≥3,000 MET-min/week), minimally active (≥600 MET-min/week or equivalent activity frequency without meeting HEPA criteria), and inactive (below these thresholds). The minimally active category corresponds approximately to the minimum level of physical activity recommended by the WHO for substantial health benefits, namely at least 150 min/week of moderate-intensity, 75 min/week of vigorous-intensity, or an equivalent combination of moderate- and vigorous-intensity physical activity (1). Individuals classified as minimally active therefore met the recommended minimum level for health-enhancing physical activity, whereas those classified as inactive did not. Sociodemographic and psychological covariates, including sex, age, monthly income, duration of residence, PHQ-9 scores, and life satisfaction, were also measured, and the results are presented in Table 2.

**Table 2:** Measurement and descriptive statistics of study variables (*N* = 555).

Category	Variable	Scale/Categories	*N* (%) or Mean (SD)
Dependent variable	PA level	Inactive level	85 (15.3%)
		Minimally active level	379 (68.3%)
		HEPA level	91 (16.4%)
Sociodemographic variable	Gender	Male	193 (34.8%)
		Female	362 (65.2%)
	Age	Continuous	43.50 (14.23)
	Monthly income	<3,000,000 KRW	178 (32.1%)
		3,000,000 – 5,000,000 KRW	221 (40.3%)
		≥5,000,000 KRW	156 (28.5%)
	Duration of residence	<5 years (ref)	155 (27.9%)
		5–10 years	157 (28.3%)
		10–15 years	148 (26.7%)
		15–20 years	42 (7.6%)
		≥20 years	53 (9.5%)
Psychological variable	PHQ-9	0–27	2.12 (3.05)
	Life satisfaction	1–9	7.46 (1.42)

Values are presented as N (%) for categorical variables and as mean (SD) for continuous variables. SD, standard deviation; PA, physical activity; HEPA, health-enhancing physical activity; PHQ-9, Patient Health Questionnaire-9; KRW, Korean won; ref, reference category.

### 2.4 Topography-sensitive 2SFCA

Unlike the conventional 2SFCA method that measures access along a planar distance, the T-S2SFCA model incorporates the walking burden imposed by slope into the accessibility measurement. Catchment radius $D_{r}$ was set to a pedestrian network distance of 400 m. This distance, which corresponds to an approximately 5-min walk, has been widely adopted as a reference distance in walking-based accessibility research (37, 38).

Applying the concept proposed by Satoh et al. (39), the model estimates a slope-adjusted travel distance l , in which travel costs increase nonlinearly with increasing slope (Equation 1 and Equation 2). The slope-adjusted costs of individual network links were summed along the least-cost pedestrian route between each demand and supply location to obtain the slope-adjusted travel distance used in the T-S2SFCA model. No separate slope-based cutoff was applied; instead, increasingly steep slopes were represented through the continuous increase in travel cost specified in Equation 2. Here, $h_{s}$ and $h_{e}$ represent the elevations of the start and end points of each link, respectively; $\mathrm{\Delta h}=h_{e}-h_{s}$ represents elevation difference; d denotes horizontal distance; θ indicates slope angle; and l represents slope-adjusted travel distance. For example, a 100 m horizontal network link with an absolute slope angle of 10° corresponds to a slope-adjusted travel distance of approximately 119.2 m (Appendix Figure A).

$$\theta =\arctan(\frac{\mathrm{\Delta h}}{d})$$ (1)

$$l=\frac{d}{\cos\theta }(1+\text{sinθ})$$ (2)

The T-S2SFCA model comprises two steps. First, for each supply location j , all demand locations i within the catchment radius $D_{r}$ are included to calculate the supply-to-demand ratio $R_{j}$ (Equation 3). Demand locations are not simply aggregated; rather, they are converted into weighted demand values using a distance-decay function, where $SV_{j}$ represents the supply volume at supply location j . Based on previous studies, the number of facilities of the same type was used as the supply volume, whereas facility area was used for public green spaces (40). $P_{i}\;$ denotes the population size of demand location i and $c_{\mathrm{ij}}$ represents the slope-adjusted travel distance between demand location i and supply location j . By incorporating population demand within each facility catchment, the T-S2SFCA model accounts for competition among residents who may share the same facilities rather than treating facility supply as independently available to each resident.

$$R_{j}=\frac{SV_{j}}{\sum_{i\in (c_{\mathrm{ij}}\leq D_{r})}P_{i}f(c_{\mathrm{ij}})}$$ (3)

The distance-decay function $f(c_{\mathrm{ij}})$ was modeled using a Gaussian function such that the accessibility contribution of a supply location decreases continuously as the travel distance increases within the threshold distance $D_{r}$ (Equation 4). Contributions beyond the threshold distance were assigned a value of zero. The robustness of the 400 m catchment radius was confirmed across alternative radii of 300–700 m (Appendix Table B1 and Appendix Figure B1).

$$f(c_{\mathrm{ij}})=\{\begin{array}{c} \frac{e^{-\frac{1}{2}{\left(\frac{c_{\mathrm{ij}}}{D_{r}}\right)}^{2}-}e^{-\frac{1}{2}}}{1-e^{-\frac{1}{2}}},c_{\mathrm{ij}}\leq D_{r}\\ 0,\;\;c_{\mathrm{ij}}>D_{r}\;\; \end{array}$$ (4)

In the second step, for each demand location i , the supply-to-demand ratios $R_{j}$ of all supply locations j within the catchment radius $D_{r}$ were aggregated using the distance decay weight to estimate the accessibility index $A_{\mathrm{ik}}$ for facility type k (Equation 5) (41).

$$A_{\mathrm{ik}}=\sum_{j\in \{c_{\mathrm{ij}}\leq D_{r}\}}R_{j}f(c_{\mathrm{ij}})$$ (5)

$$CA_{i}=\sum_{k=1}^{K}w_{k}\cdot A_{\mathrm{ik}}$$ (6)

The accessibility estimates calculated for each facility type were integrated into a composite accessibility index $CA_{i}$ by applying differential weights $w_{k}$ derived from an Analytic Hierarchy Process survey conducted with 40 experts in the field of urban planning (Appendix Table C), where $CA_{i}$ represents the composite accessibility index for demand location i , $A_{\mathrm{ik}}$ denotes the accessibility estimate for facility type k , $w_{k}$ indicates the corresponding weight, and K represents the total number of facility types (Equation 6).

## 3 Results

### 3.1 Spatial mismatch between topographic characteristics and ALI supply in Seongnam

In Seongnam, distinct spatial differences in composite ALI accessibility were observed depending on slope conditions (Table 3). Approximately one-fifth of the total population resided in high-slope grid cells with a gradient of 10° or more, and the difference in accessibility estimates between the conventional 2SFCA and T-S2SFCA models widened as the slope increased. This suggests that the accessibility of a substantial number of residents may be misrepresented when measured using planar distance alone. To examine these differences more specifically, the spatial distribution of the composite ALI accessibility and accessibility patterns by facility type were examined.

**Table 3:** Composite ALI accessibility estimates from 2SFCA and T-S2SFCA models by slope condition.

Slope condition	Population share (%)	2SFCA composite accessibility Mean (SD)	T-S2SFCA composite accessibility Mean (SD)	Decrease from 2SFCA to T-S2SFCA (%)
<5°	53.6	0.0742 (0.1344)	0.0625 (0.1179)	15.8
5–10°	25.5	0.0485 (0.0964)	0.0386 (0.0749)	20.4
≥10°	20.8	0.0275 (0.0657)	0.0206 (0.0454)	25.1
Total	100.0	0.0579 (0.1154)	0.0476 (0.0979)	17.8

SD, standard deviation; ALI, active living infrastructure; 2SFCA, two-step floating catchment area; T-S2SFCA, topography-sensitive two-step floating catchment area.

**Figure 2:**
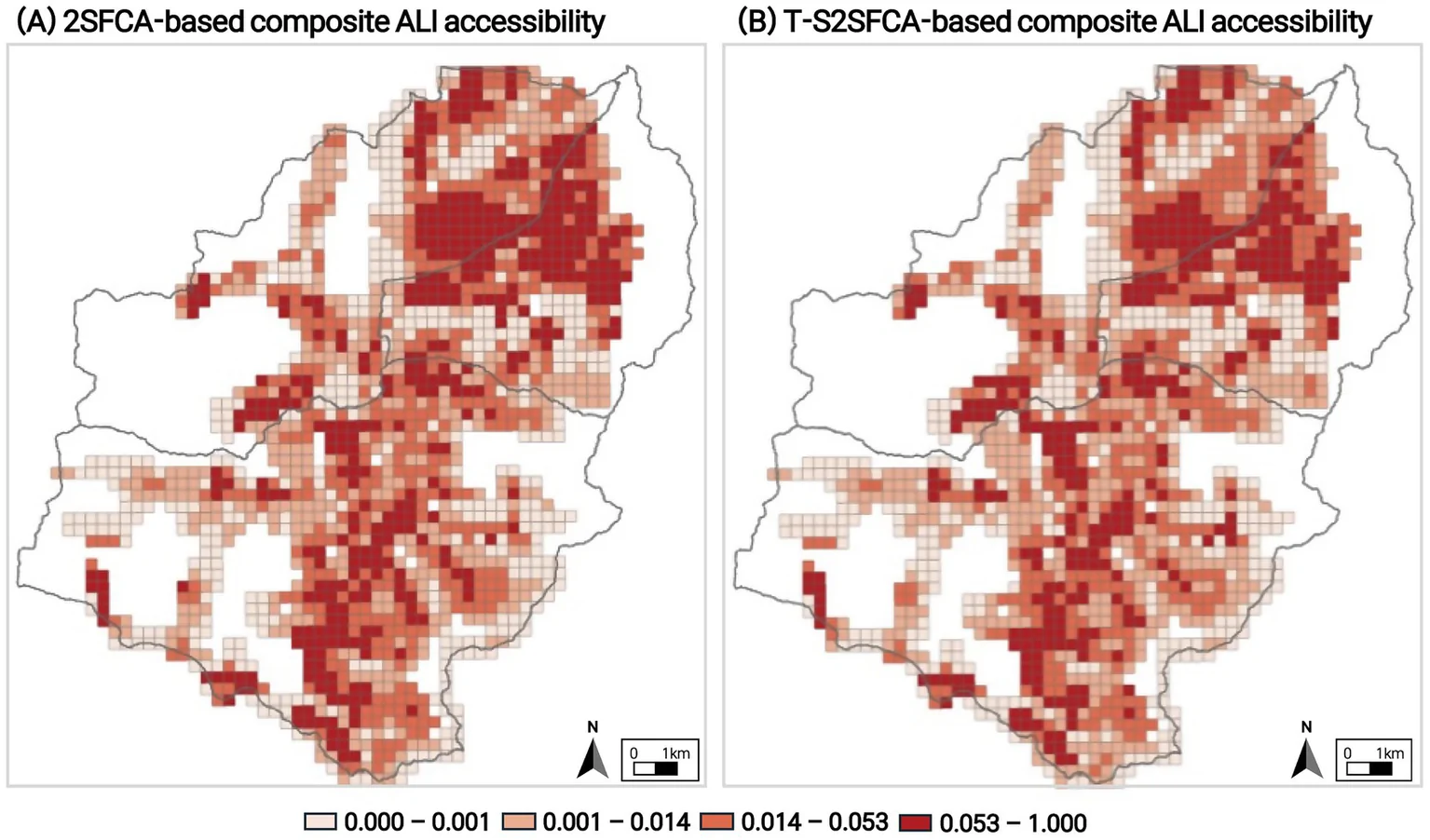
**(A)** 2SFCA-based composite ALI accessibility; and **(B)** T-S2SFCA-based composite ALI accessibility.

The decrease in composite ALI accessibility after topographic correction was concentrated in the steeply sloped residential areas of the northern part of the city (Figure 2). Under the conventional 2SFCA model, which measured access along planar distance, high-accessibility areas appeared widespread regardless of terrain. Once the walking burden of slope was incorporated in the T-S2SFCA model, however, accessibility declined substantially in the northern sloped areas, whereas it remained largely unchanged in the flat southern areas, so that the spatial extent of high-accessibility areas contracted toward gentler terrain. On average, the conventional 2SFCA model estimated composite ALI accessibility to be approximately 22% higher than the T-S2SFCA model, and this difference widened to approximately 33% in high-slope grid cells with slopes of 10° or greater. This evidence confirms that conventional 2SFCA measures overestimate neighborhood accessibility by ignoring vertical terrain and mask the actual walking constraints embedded in the steeply sloped residential areas.

These accessibility gaps varied according to the facility type (Table 4). Accessibility to sports facilities in steeply sloped areas was only one-fifth that in flat areas, whereas accessibility to retail facilities and public transit was less than half that observed in flat areas. By contrast, the gap for public green spaces was less than 2.9%, which was the smallest among the facility types. This is because, unlike most facilities that are concentrated in flat central areas, public green spaces are distributed close to steeply sloped residential areas in the form of mountains and large green spaces.

**Table 4:** Facility-specific T-S2SFCA accessibility differences between low-slope and high-slope grid cells.

Facility type	Mean accessibility in <5° grids	Mean accessibility in ≥10° grids	Change from <5° to ≥10° grids (%)
Retail facility	0.8919	0.3357	−62.4
Sports facility	0.0702	0.0130	−81.5
Community facility	0.0576	0.0421	−26.9
Public transit	0.0612	0.0253	−58.7
Public green space	646.886	628.214	−2.9

Public Green space accessibility was calculated using area-based supply, whereas other facility types were calculated using count-based supply. T-S2SFCA, topography-sensitive two-step floating catchment area.

In summary, the steeply sloped residential areas in Seongnam accommodate a substantial population yet have low accessibility to ALI facilities. Overall, a spatial mismatch was identified, in which the facility supply was concentrated in flat areas, whereas the pedestrian burden was concentrated in sloped areas. This mismatch was not fully revealed by planar-distance-based measurements, and its scale and distribution became apparent only after topographic correction.

### 3.2 Associations between T-S2SFCA-based ALI accessibility and PA levels

Two multinomial logistic regression models were constructed to distinguish the relationships among facility-type, composite ALI accessibility, and PA levels. Model 1 included facility-type accessibility indicators to examine the independent associations of individual ALI components, whereas Model 2 included a composite accessibility index to evaluate the integrated association of ALI accessibility. All accessibility variables were standardized to z-scores after log(x + 1) transformation. Thus, the odds ratio represents the change in the odds of being minimally active or achieving HEPA relative to being inactive per standard deviation increase in accessibility. Variance Inflation Factors (VIF) were assessed for all predictors included in the regression models, including the accessibility variables and covariates. All VIF values were below 10, indicating no substantial multicollinearity.

Distinct patterns related to PA levels were observed for individual characteristics (Table 5). Higher life satisfaction was associated with approximately 1.2 to 1.4 times higher odds of being minimally active and achieving HEPA. Respondents who had lived in the same area for 15 years or longer had more than four times the odds of achieving HEPA than those with less than 5 years of residence, whereas women had approximately half the odds of achieving HEPA than men.

**Table 5:** Multinomial logistic regression results for T-S2SFCA-based ALI accessibility and PA levels.

Variables		T-S2SFCA							
		Model1: Facility-specific				Model2: Composite Index			
		Minimal vs Inactive		HEPA vs Inactive		Minimal vs Inactive		HEPA vs Inactive	
		OR	95% CI	OR	95% CI	OR	95% CI	OR	95% CI
Gender	Male	References							
	Female	0.636	0.361–1.121	0.438*	0.214–0.893	0.625	0.357–1.108	0.469*	0.234–0.941
Age (continuous)		1.007	0.986–1.028	1.009	0.984–1.035	1.006	0.986–1.026	1.009	0.985–1.034
Monthly income	<3,000,000 KRW	References							
	3,000,000 – 5,000,000 KRW	0.841	0.435–1.572	1.189	0.523–2.702	0.837	0.445–1.576	1.150	0.514–2.577
	≥5,000,000 KRW	0.815	0.381–1.543	1.133	0.412–2.383	0.754	0.381–1.492	0.939	0.398–2.216
Duration of residence	<5 years	References							
	5–10 years	0.856	0.429–1.706	1.983	0.758–5.189	0.863	0.438–1.702	1.985	0.769–5.122
	10–15 years	0.447*	0.234–0.855	1.795	0.626–4.006	0.464*	0.244–0.882	1.627	0.652–4.062
	15–20 years	0.957	0.319–2.871	4.522*	1.100–17.004	1.005	0.339–2.981	4.388*	1.142–16.870
	≥20 years	1.261	0.394–4.031	5.047*	1.424–22.074	1.228	0.387–3.895	5.452*	1.414–21.025
PHQ-9		1.062	0.965–1.168	1.068	0.945–1.206	1.064	0.968–1.170	1.072	0.951–1.209
Life Satisfaction		1.226*	1.007–1.492	1.437**	1.093–1.850	1.217*	1.001–1.479	1.399*	1.081–1.810
Retail facility accessibility		1.550*	1.017–2.465	1.741*	1.031–2.940				
Community facility accessibility		1.304	0.921–1.846	1.414*	1.041–2.124				
Public Green Space accessibility		1.389*	1.049–1.839	2.110***	1.582–3.277				
Sports facility accessibility		0.858	0.454–1.691	1.309*	1.059–1.614				
Public Transit accessibility		0.843	0.578–1.229	0.826	0.532–1.271				
Composite Accessibility						1.594**	1.173–2.164	2.115***	1.484–3.001
Pseudo R^2^ (Nagelkerke)		0.184				0.138			
LLR *p*-value		<0.0001				<0.0001			

*Significance at 95%; **significance at 99%; ***significance at 99.9%. Odds ratios and 95% confidence intervals are reported. Inactive was used as the reference category for PA level. OR, odds ratio; CI, confidence interval; PA, physical activity; HEPA, health-enhancing physical activity; PHQ-9, Patient Health Questionnaire-9; LLR, likelihood-ratio test; T-S2SFCA, topography-sensitive two-step floating catchment area.

Facility type was also associated with different PA levels. In Model 1, the facility type most strongly associated with PA level was public green space, which was associated with approximately 1.4 times higher odds of being minimally active and more than twice the odds of achieving HEPA. Retail facility accessibility was also associated with both levels, with approximately 1.6- and 1.7-times higher odds of being minimally active and achieving HEPA, respectively. In contrast, sports and community facility accessibility was associated only with HEPA, with approximately 1.3- and 1.4-times higher odds, respectively.

In Model 2, composite ALI accessibility was significantly associated with both PA levels. Higher composite ALI accessibility was associated with approximately 1.6 times higher odds of being minimally active and 2.1 times higher odds of achieving HEPA relative to being inactive. This suggests that an environment supporting PA is shaped not by accessibility to a single facility, but by the way multiple ALI components are integrated, and supports the empirical validity of the composite accessibility index that operationalizes ALI as an integrated concept. To isolate the contribution of the topographic correction, the same model was additionally estimated with the composite index derived from the conventional 2SFCA method (Appendix Table D), so that the two specifications differ only in whether the accessibility measure accounts for slope. Under the conventional specification, composite accessibility was not significantly associated with either activity level, and model fit was weaker, with the Nagelkerke pseudo-*R*^2^ at 0.094 compared with 0.138 for the T-S2SFCA-based specification and the likelihood ratio test at *p* = 0.0034 compared with *p* < 0.0001. Accounting for slope thus contributed to capturing the differences in accessibility associated with PA.

## 4 Discussion

This study measured ALI accessibility in Seongnam by integrating five facility types into a single composite index and examining their relationship with PA levels. The central finding is that ALI accessibility is best understood not as proximity to any single facility, but as a neighborhood’s combined capacity to support daily activities. Composite ALI accessibility was associated with both minimally active and HEPA levels, and facility-type results showed that this combined capacity was assembled from components that played distinct yet complementary roles.

The differing associations across facility types suggest that ALI may be related to PA through multiple pathways. Public green spaces showed the strongest association with HEPA, while also being associated with minimally active status, reflecting their multifunctional role as resources for walking, exercise, leisure, and rest (13, 16). The association of retail facilities with both activity levels may reflect their role as everyday destinations, where repeated visits are accompanied by the accumulation of short walking trips (30, 37). Sports and community facilities, in contrast, were associated only with HEPA, a pattern that may reflect their role as exercise-oriented venues and program-based hubs for intentional, structured activity. This interpretation fits the Korean context, where community facilities often host organized programs, such as health classes and group exercises.

These distinct roles do not imply that the facilities operate in isolation. Daily walking rarely consists of a round trip to one destination; it accumulates within a chain of trips connecting several facilities, such as passing through retail areas on the way to public green spaces (42). Public green spaces and retail were associated with overall participation, whereas sports and community facilities were associated specifically with health-enhancing activities, which is consistent with facilities functioning as complementary components of trip chains rather than as separate resources. Existing research has largely evaluated these facilities as separate destinations (14, 43), without considering how they combine with residents’ daily movements. The ALI perspective addresses this by conceptualizing diverse built environment elements as a single interdependent system, enabling the evaluation of a neighborhood’s overall capacity to support PA beyond the simple aggregation of individual facilities. This integrated view reframes neighborhood facilities not as a list of separate amenities, but as a connected system through which residents’ everyday trips translate into PA.

However, detecting this integrated capacity depends on how accessibility is measured. When accessibility was estimated using the conventional 2SFCA method, composite accessibility was not associated with PA levels, whereas the topography-adjusted measure was associated with both minimally active and HEPA. This indicates that accounting for the walking burden imposed by slope brings the composite index closer to the accessibility residents actually experience, enabling the association with composite ALI accessibility to be detected where planar distance alone could not.

The accessibility measure used here is designed to reduce, though not eliminate, the neighborhood-level dependence that motivates this concern. The T-S2SFCA measure is computed at each participant’s residential location, with catchments delineated outward from that location rather than bounded by predefined zones, and facility supply weighted by the population demand competing for it. Accessibility therefore varies continuously across space, and the topography adjustment further differentiates residents whose walking conditions differ despite living in close proximity.

We acknowledge, however, that this does not fully address dependence arising from unobserved neighborhood attributes such as perceived safety or social cohesion. We have added this point to the limitations section, noting that a multilevel specification with a sufficient number of sampled neighborhoods would allow such dependence to be modeled directly.

This study had some limitations. First, PA was measured using the self-reported IPAQ, which may be subject to recall bias, overreporting, and measurement error; modeled walking routes and slope estimates may also be affected by uncertainty in the OpenStreetMap pedestrian network and 5 m DEM. Second, because the sample was recruited through an online survey, certain groups such as older adults may have been underrepresented. Third, because this study was conducted in a single city, the generalizability of the results to cities with different terrain, urban density, or transport systems may be limited, and validation across diverse urban contexts is needed. Fourth, the cross-sectional design does not permit causal inference and cannot rule out residential self-selection, reverse causality, or unmeasured confounding. In addition, the regression models did not explicitly account for potential neighborhood-level clustering or spatial dependence arising from unobserved neighborhood attributes. Future studies should address these issues using longitudinal designs and, with a sufficient number of sampled neighborhoods, multilevel models. Despite these limitations, this study presents an analytical framework that evaluates ALI accessibility by combining a composite, multi-facility measure with slope-adjusted walking costs and links it to IPAQ-based PA levels.

## 5 Conclusion

This study measured the ALI accessibility in Seongnam by integrating multiple facility types into a single composite index and examining their relationship with PA levels. By conceptualizing diverse neighborhood facilities as a single interdependent infrastructure rather than as a set of separate destinations, the analysis showed that composite ALI accessibility was associated with both minimally active and HEPA and that this combined capacity was assembled from facility types that played distinct yet complementary roles. Public green spaces and retail facilities were associated with both activity levels, whereas sports and community facilities were associated with HEPA only, with public green spaces showing the strongest association with health-enhancing activities. These distinct roles are consistent with the way everyday walking accumulates across connected destinations, rather than within a single facility. Notably, this combined capacity emerged only when accessibility accounted for the walking burden of the slope, indicating that how access is measured determines whether the integrated role of ALI could be observed.

These findings have practical implications in urban planning. Because a neighborhood’s capacity to support PA arises from how multiple facilities work together rather than from any single facility, planning should treat ALI as an integrated system rather than adopting a facility-by-facility approach. To this end, rather than examining the supply of each facility type in isolation, planning should coordinate facility placement and pedestrian route improvements based on how effectively retail, public green spaces, sports, and community facilities are integrated into a single accessibility system within a neighborhood. In improving pedestrian routes, priority should be given to areas where facilities are nearby yet remain difficult to reach on foot because of steep slopes, thereby narrowing the gap between facility supply and effective walking access. Through planning that considers facility composition and the pedestrian environment together, ALI can more effectively support inclusive and sustained PA across diverse neighborhoods.

## Data Availability

The original contributions presented in the study are included in the article/Supplementary material, further inquiries can be directed to the corresponding author/s.
