# Supplementary material for: A topography-sensitive 2SFCA approach to measuring accessibility to active living infrastructure and its association with health-enhancing physical activity
Source: Front Public Health. 2026 Sep 4;14:1923137. doi: 10.3389/fpubh.2026.1923137 (PMC13614116; doi:10.3389/fpubh.2026.1923137)
Supplement: Supplementary file 1 [file Data_Sheet_1.PDF]

Appendix

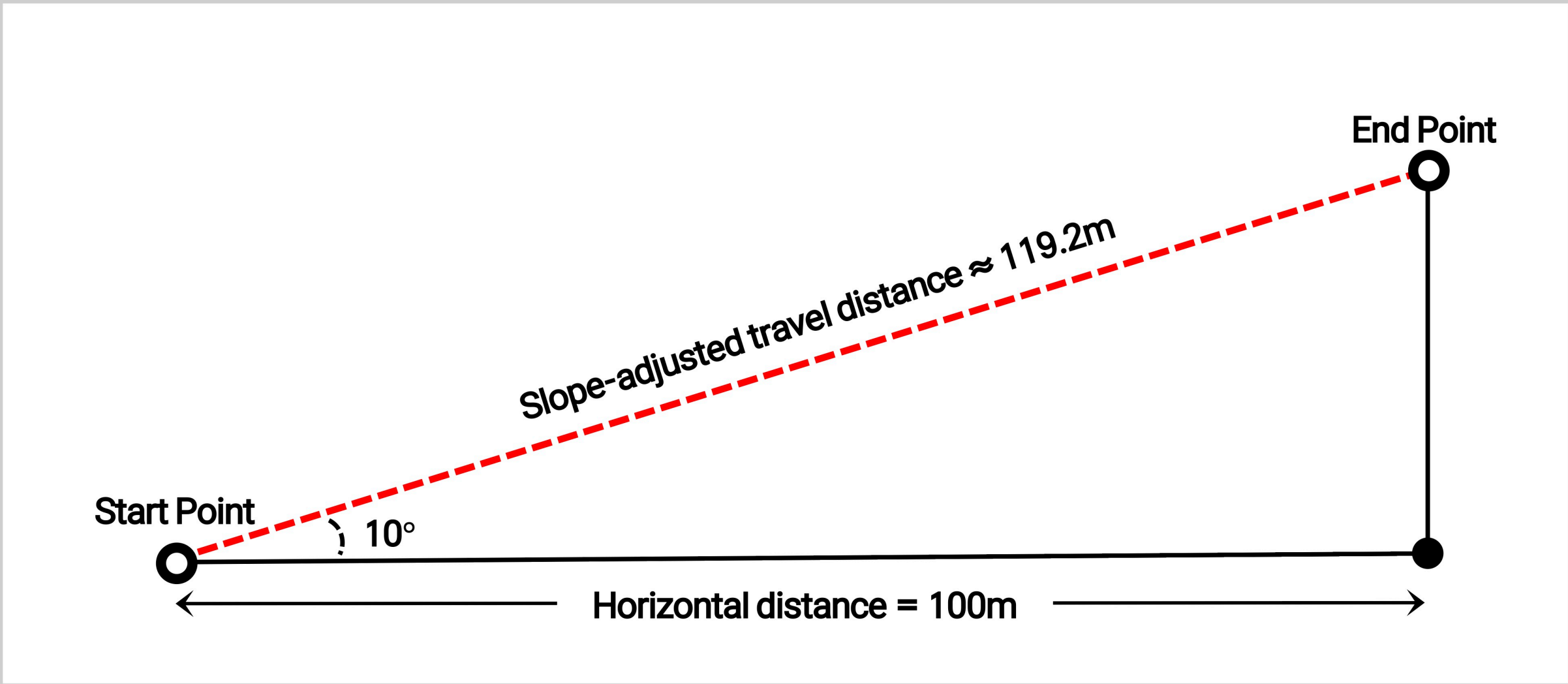

Figure A. Schematic illustration of slope-adjusted travel distance in the T-S2SFCA model

To assess whether the results were sensitive to the choice of catchment radius, composite ALI accessibility was recalculated at radii ranging from 300 to 700 m in 100 m increments, and its association with the IPAQ continuous score (MET-minutes per week) was estimated at each radius using the same covariates as in the main analysis. The association remained positive and statistically significant across all radii and was strongest at 400 m, supporting the use of 400 m in the main analysis (Table A1, Figure A1).

Table B1. Robustness Check of the Association between Composite ALI Accessibility and the IPAQ Continuous Score across Catchment Radii (300–700 m)

Catchment Radius	Regression Coefficient	P-value	95% Confidence Interval
300m	0.122*	.004	[0.039, 0.205]
400m	0.138**	.001	[0.056, 0.220]
500m	0.120*	.010	[0.029, 0.211]
600m	0.108*	.010	[0.026, 0.190]
700m	0.081*	.014	[0.017, 0.145]

Figure B1. Robustness check of composite ALI accessibility across catchment radii (300–700 m)

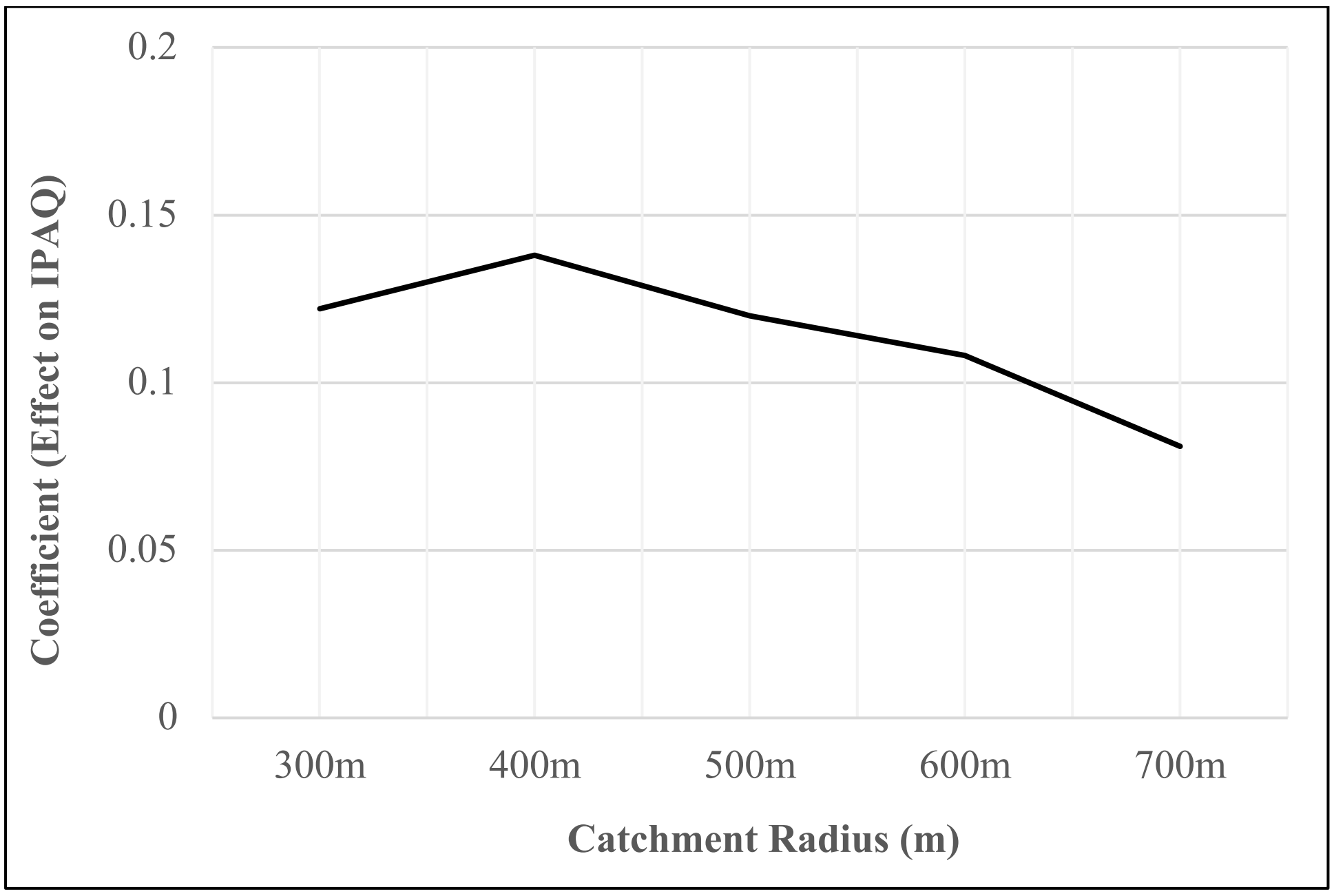

Table C. AHP-Derived Weights for ALI Facility Types

**ALI facility type**	**Weight**
Retail facilities	0.29
Public transit	0.25
Public green spaces	0.19
Sports facilities	0.15
Community facilities	0.12
**Total**	**1.00**

Table D. Multinomial Logistic Regression Results for Conventional 2SFCA-based Composite ALI Accessibility and PA Levels

Variables		2SFCA			
		Model 2: Composite Index			
		Minimal vs Inactive		HEPA vs Inactive	
		OR	95% CI	OR	95% CI
Gender	Male	**References**			
	Female	0.654	0.376–1.138	0.562	0.284–1.109
Age (continuous)		1.004	0.985–1.023	1.008	0.985–1.032
Monthly income	<3,000,000 KRW	**References**			
	3,000,000 – 5,000,000 KRW	0.746	0.403–1.381	0.98	0.448–2.148

Variables		2SFCA			
		Model 2: Composite Index			
		Minimal vs Inactive		HEPA vs Inactive	
		OR	95% CI	OR	95% CI
	≥5,000,000 KRW	0.757	0.388–1.480	0.960	0.415–2.221
Duration of residence	<5 years	**References**			
	5–10 years	0.844	0.430–1.657	2.062	0.805–5.281
	10–15 years	0.433*	0.225–0.833	1.652	0.663–4.117
	15–20 years	0.919	0.310–2.719	3.628	0.964–13.646
	≥20 years	1.384	0.439–4.364	6.079**	1.602–23.072
PHQ-9		1.083	0.983–1.192	1.085	0.963–1.223
Life Satisfaction		1.224*	1.011–1.482	1.385**	1.079–1.778
Composite Accessibility		1.112	0.871–1.419	0.875	0.636–1.202
Pseudo R^2 (Nagelkerke)		0.094			
LLR p-value		0.0034			

Note. Odds ratios with 95% confidence intervals are reported, with Inactive as the reference category. Accessibility was standardized as log(x+1) followed by z-score transformation.
